# Self-management and information needs of adults with seasonal allergic rhinitis in the Netherlands: A focus group study

**DOI:** 10.1177/13591053241272150

**Published:** 2024-08-22

**Authors:** Bob C Mulder, Marise J Kasteleyn, Lisbeth Hall, Arnold JH van Vliet, Letty A de Weger

**Affiliations:** 1 Wageningen University & Research, The Netherlands; 2 Leiden University Medical Center, The Netherlands; 3 National Institute for Public Health and the Environment (RIVM), The Netherlands

**Keywords:** focus groups, information needs, seasonal allergic rhinitis, self-management

## Abstract

This focus group study explored the needs, preferences and beliefs of adults with seasonal allergic rhinitis regarding their self-management practices, and related information use and needs. Four focus groups were held, two online and two on location. The 20 participants (11 women); *M*_age_ = 39.0 years (range: 21–56 years) were reluctant to identify themselves as patients, trivializing their complaints while avoiding being confronted too much with their condition. Participants often expressed low trust in the effectiveness of medication and the ability of healthcare to alleviate their complaints. This resulted in relatively low openness to information such as personalized pollen predictions. Findings were synthesized under three interrelated themes: ‘Being ill, but not a patient: it’s bad, but you learn to live with it’, ‘Individual search for what does or doesn’t work’ and ‘Information needs and sources’. Implications for communication supportive of self-management practices for seasonal allergic rhinitis are discussed.

## Introduction

### Societal relevance

Seasonal allergic rhinitis due to pollen allergy is a common condition affecting 20%–30% of the population in north-western Europe (Leth-Møller et al., 2020; Savouré et al., 2023). One of the most important causes of allergic rhinitis is pollen from wind-pollinated plants. Often people with allergic rhinitis do not go to the doctor with their complaints. As a result, the disease is under-diagnosed (Maurer and Zuberbier, 2007). Nevertheless, the symptoms can have major impacts on daily functioning although the impacts can be trivialized by both people suffering from it and physicians (Marple et al., 2007; Muzalyova et al., 2019). Seasonal allergic rhinitis can significantly reduce the quality of life (Meltzer, 2001; Rosario et al., 2021) and the condition can lead to lost working days (Bhattacharyya, 2012) and reduced work and study performance (Bhattacharyya, 2012; Blaiss et al., 2018; Walker et al., 2007). Due to the above factors and the high prevalence of allergic rhinitis, the burden of disease is considerable (Colás et al., 2017). The financial and societal costs are also sizeable with the indirect costs (lost working days, reduced work performance) being higher than the direct costs of medical care (Colás et al., 2017).

There has been a sharp increase in allergic respiratory diseases in recent decades (Xie et al., 2020). The cause of this increase is complex and many factors likely play a role, such as increased hygiene, increased antibiotic use, changes in lifestyle and eating habits and air pollution (Smits et al., 2016). Climate change may also contribute to this increase. Rising CO_2_ concentrations and higher temperatures due to climate change are leading to changes in the onset, duration and intensity of the pollen season, and shifts in the distribution of allergenic species (de Weger et al., 2021; Frei, 2020; Hoebeke et al., 2018; Lind et al., 2016). Changing climatic conditions can also be favourable for the establishment of non-native allergenic species (Lake et al., 2017). In addition, there is some evidence that increasing CO_2_-concentrations may change the allergenicity of pollen (El Kelish et al., 2014). The aforementioned changes are expected to increase the incidence and prevalence of seasonal allergic rhinitis (pollen allergy).

The treatment of seasonal allergic rhinitis is mainly symptomatic and requires continuous self-management, for example, through saline irrigation of the nose or over-the-counter medication. Despite the importance of taking medication regularly and on time, many people do not adhere to this or prefer not to take any medication (Cvetkovski et al., 2018; Muzalyova et al., 2019). Allergen avoidance behaviours, such as staying indoors, wearing sunglasses or face masks, can reduce exposure, but doing so is impractical for many persons (Marple et al., 2007). Therefore, it seems that many people manage their symptoms sub-optimally. Better support and information may help increase their capacity for self-management.

### Scientific relevance

A few studies using qualitative approaches have examined people’s capacity to manage their pollen allergy symptoms (Cvetkovski et al., 2018; Muzalyova et al., 2019). Although seasonal allergic rhinitis is perceived as having an impact on their quality of life, through its effect on daily activities, work performance and social life, people strongly believe they can manage it themselves (Cvetkovski et al., 2018; Muzalyova et al., 2019). Nevertheless, a number of issues have been identified which affect the self-management of allergic rhinitis. Delayed diagnosis and misdiagnosis can lead to treatment fatigue and also contribute to a people’s confidence in their ability to manage their allergic rhinitis themselves, perceiving self-management as a more efficient use of time than healthcare appointments (Cvetkovski et al., 2018). This strong belief in self-management contributes to its burden on the sufferer as it widens the distance to healthcare providers, thus decreasing access to the latest guidelines and treatment options (Cvetkovski et al., 2018). Furthermore, the focus of self-management is predominantly on medication rather than avoidance, but medication costs can affect self-management choices as do certain beliefs which oppose medication use (Cvetkovski et al., 2018). Regarding avoidance strategies, Muzalyova et al. (2019) found that although most participants in their survey were aware of the most common strategies, they only applied half of them. Interestingly, participants disliked being regarded as having an illness and felt that their symptoms, such as sneezing, were often misinterpreted and stigmatized (Cvetkovski et al., 2018). This must particularly have been the case during the COVID pandemic.

As access to local and relevant pollen information is expected to empowerment and self-management, Medek et al. (2019) studied the perceived benefits of this information through a questionnaire. Those who already had access to local pollen information (50% of respondents) were unanimous in finding it useful, with the main reasons given being allergen avoidance, medication decisions and preparation and planning. Nine out of ten participants without access to this information indicated that they would want this service, with their reasons being similar to the group which already had access, namely, allergen avoidance, forecasting symptoms or pollen levels and medication decisions (Medek et al., 2019).

From the above, it is clear that providing accurate information on when, where and which pollen types occur in the air is a key factor in helping reduce pollen-related symptoms. In doing so, it is important to identify information needs and how the information should be presented to influence behaviour as effectively as possible. As seasonal allergic rhinitis is predominantly a self-managed condition, the kind of information and the manner it is offered need to match self-management practices and current beliefs in order to be effective. Although, for reasons mentioned above, people strongly believe in their self-management, many nevertheless manage their symptoms sub-optimally. In the light of increasing possibilities for accurately predicting local pollen concentrations, an in-depth understanding of the current role of knowledge and information in self-managing seasonal allergic rhinitis is required as well as a better understanding of the related latent information needs.

The aim of this study is to explore current self-management practices, beliefs and the role of knowledge and information among adults with seasonal allergic rhinitis and to understand their (current and latent) information needs and preferences, particularly regarding personalized and forecast local pollen concentration information. This is increasingly relevant given that climate change is already leading to rising pollen concentrations and a change in the timing and duration of the pollen seasons in many countries.

## Methods

### Choice of methodology

A focus group study was designed, as focus groups enable exploring views and experiences, in particular because participants can exchange anecdotes and ask each other questions, thus revealing not only what they think, but also how and why (Kitzinger, 1995).

### Recruitment

Participants for the four focus groups were recruited in two ways: a commercial research agency recruited participants for two focus groups, and a pharmacy network allowed the research team to recruit participants for the other two focus groups. All participants were offered €55, – as a financial incentive to take part.

The commercial research agency used purposeful sampling to select participants from their own database of over 25,000 people. One group was compiled of people who stated having severe seasonal allergenic complaints, while the other group was recruited to comprise people who stated having mild complaints. Across both groups, the aim was to recruit a mix of participants regarding age, gender, educational level and residence (i.e. throughout the Netherlands). The recruitment agency was instructed to recruit six participants for each of the two online focus groups. Shortly before each focus group, the researchers received a list of six participants and one reserve participant, including their demographics and contact details. On both occasions, one of the participants did not show up in the online meeting, and the reserve participant was called and invited to participate.

A research assistant recruited participants via two pharmacies, by calling adults who received their pollen allergy medication through those pharmacies. One pharmacy was located in a mid-sized university town in a central region of the Netherlands, while the other pharmacy was located in a small town in the east of the Netherlands. Except for place of residence, recruitment again aimed to include a mix of participants, in terms of demography as well as the severity of their allergenic complaints. Again aiming for a group size of six participants, the research assistant recruited 11 participants from each town, taking into account potential drop-out or no-shows.

### Participants

The four focus groups totalled 20 participants, with three focus groups having six participants and one group having two participants, because these were the only ones to show up from a group of nine people who had agreed to participate. Of the 20 participants, 11 were female, and age ranged from 21 to 56 years (*M* = 39.0; SD = 11.4). See Table 1 for an overview of participant characteristics.

**Table 1.** Participant characteristics.

Focus group*	Gender	Age (years)	Educational level	Allergenic complaints
1	Female	40	Higher vocational	Moderate to severe
1	Female	53	Intermediate vocational	Severe
1	Female	50	Higher vocational	Severe
1	Female	28	Intermediate vocational	Severe
1	Male	40	Higher vocational	Severe
1	Male	33	Lower vocational	Severe
2	Male	46	University	Mild
2	Male	47	Higher vocational	Mild to moderate
2	Male	51	Higher vocational	Mild to moderate
2	Female	48	Intermediate vocational	Mild
2	Female	54	Higher vocational	Mild to moderate
2	Male	30	University	Mild to moderate
3	Male	38	University	Severe
3	Female	23	University	Severe
4	Female	26	Intermediate vocational	Severe
4	Female	28	Lower vocational	Severe
4	Male	56	Higher vocational	Moderate to severe
4	Male	22	University	Moderate
4	Female	21	Intermediate vocational	Severe
4	Female	46	Intermediate vocational	Mild

* Focus groups 1 and 2 were recruited by a research agency, and held online; whereas focus groups 3 and 4 were recruited by the research team via two pharmacies, and held on location.

### Data collection

In May and June 2022, four focus groups were conducted, each lasting between approximately 50 minutes and 2 hours. Focus groups 1 and 2 (recruited by the research agency) were held online because of the geographic spread of the participants. Online meetings took place via Microsoft Teams, which were recorded after all participants gave their consent to start the recording. Focus groups 3 and 4 were held on location in the towns from where the participants were recruited (i.e. also where the pharmacies were located), and were audiotaped using a voice recorder.

The first author (BM) moderated all four focus groups, using a topic list (see Supplemental Appendix I) that started by asking participants to draw on paper everything they were allergic for. The topic list was based on both literature and brainstorm and feedback rounds with the research team. After the drawing exercise, each participant explained what s/he had drawn, while the moderator asked how long the participant had experienced these allergies, and how the participant had found out. This ‘warming up’ round helped introduce everyone, and already led participants to ask each other questions, and/or express recognition of experiences. It also provided basic information on whether participants had other allergies besides pollen; on the type and severity of complaints; and how much participants knew about their own allergies. Proceeding from there, the opening question was ‘How have you experienced the pollen season so far?’ Subsequent topics that were introduced, mostly in the form of follow-up questions, were: knowledge of specific pollen and plants or trees participants were allergic to; severity and impact of complaints on daily life; prevention and treatment of complaints, including avoidance of pollen and medication use; and finally focusing on information needs: preferences for and perceived trustworthiness of information sources, such as pollen forecasts; needs and preferences for tailored pollen information; and motivation to fill in symptom scores in order to receive tailored information.

### Analysis

Focus group recordings were transcribed verbatim by a typist agency, and then coded using Atlas.ti (version 22). During transcription, privacy-sensitive information (e.g. names were left out). Authors BM and MK independently coded all transcripts. A set of deductive codes was derived from the topic list, and presented the starting point for both coders. Additional codes were added inductively during the coding process. Coding was seen as an intermediary step towards finding overarching themes, in line with Kiger and Varpio (2020). BM developed a thematical structure to organize and categorize recurrent and common themes in the interviews. The purpose was to identify and highlight essential elements within the collected data. BM collaborated with MK to discuss and refine the thematic structure until they reached a consensus on its content.

### Ethical approval

The study protocol was assessed and approved by the Social Sciences Ethics Committee of Wageningen University under number 2022-61-Mulder. All participants signed the informed consent after they were informed about the aim of the research, the voluntary and confidential nature of participation, and the anonymous processing of data throughout the analysis and reporting of the study.

## Results

The focus groups were conducted in a positive and open atmosphere, fostering a sense of comfort and trust. There were moments of surprise as individuals learned from one another, exchanging insights and perspectives. The discussion confirmed the challenges of anticipating and managing AR symptoms, as well as attempting to control them. Empathy was evident among participants, who shared their experiences and supported each other in dealing with symptoms and understanding the overall impact of allergies on daily life. Notably, the absence of dominant participants or signs of polarizations allowed for a rich exchange of ideas, resulting in both consensus and diverse responses.

Three main themes were identified: ‘Being ill, but not a patient: it’s bad, but you learn to live with it’, ‘Individual search for what does or doesn’t work’ and ‘Information needs and sources’. This latter theme is strongly interrelated with the previous two, as we will subsequently elaborate on.

### Theme 1: Being ill, but not a patient: it’s bad, but you learn to live with it

Most participants were diagnosed with pollen allergy in their early childhood, meaning during the primary school period. The general practitioner (GP) established the diagnosis through an allergy test. Many participants did not know the specific plant species that caused allergic reactions beyond broad categories such as ‘trees’ or ‘grass’. The low knowledge of their individual allergic profile was generally attributed to memory issues, since they had been tested such a long time ago, but many participants also said they were never tested for specific pollen allergens, or informed about their specific profile. Some participants attributed allergic complaints to experiences such as responding to mown grass, or working in the garden on specific plant species. There were also respondents who believed they were allergic to fluffy plant seeds such as dandelion or poplar.

Yes, I have the pollen too. And actually, I’ve been suffering from it for as long as I can remember. At a certain point I had a cold and it just wouldn’t go away. Anyway, so I visited the doctor for once and then it was, you just have hay fever. (focus group 3, on location)

Other allergies were common, such as for animals and house dust mites, as well as food allergies or being allergic to plasters, laundry detergents or perfumes. Some participants knew about cross-reactivity, such as between birch pollen and apples, while others were surprised to hear about it with someone recognizing the symptoms.

There was quite some variation between participants in the number and type of complaints they experienced. Most mentioned were complaints similar to having a cold, notably coughing, a runny or stuffy nose and sneezing. Itchy, dry, burning and/or tearing eyes were also very common. Although these complaints were typically evaluated as mild, these did negatively affect sleep quality and daily functioning. Severe complaints included having a fever, and shortness of breath or feeling choked. Such complaints resulted in participants having to call in sick and stay at home.

Several participants noted changes in their allergy symptoms during the course of their lives. Some observed a decrease in severity as they aged, while others mentioned a reduction in symptoms during pregnancy. Seasonal variations were also evident, with participants experiencing more symptoms on dry, windy days. Moreover, some participants reported a shift in the season that they experienced symptoms, for example in winter, whereas previously it had been limited to spring and summer. The impact of regional variation was also highlighted, as participants reported experiencing more symptoms in specific areas with distinct vegetation. However, the variation in complaints due to varying circumstances also resulted in uncertainty whether complaints could definitely be attributed to their allergy, as well as an overall sense of unpredictability regarding when their complaints would peak or not.

Sometimes I find it very apparent what it is directly related to, but sometimes I also think, ‘Well I shouldn’t really be suffering so much’, and then all of a sudden it’s terrible. (focus group 1, online)

Participants expressed a strong desire not to feel like patients and instead strive for a normal life, preferring not to be constantly confronted with their allergy status. Many of them did not identify themselves as allergy patients. On the other hand, most participants listed quite some symptoms they were experiencing, especially during the pollen season, with some of them taking daily medication all year long.

And that shortness of breath can be annoying, but once I’m somewhere else again, it’s over quickly. So I wouldn’t say it’s much of a hindrance. I know about it, so that helps. (focus group 3, on location).

This ambivalence was reflected in their views on the impact of symptoms on daily life. Most participants indicated that ‘you learn to deal with it’, which on the one hand shows acceptance, while on the other hand it also affects motivation to improve symptom management. Some participants tended to downplay their complaints, viewing them as less severe compared to being a patient requiring hospital visits.

I think, me too, but I think, if I then hear the symptoms we have and what it does sometimes. And I guess that’s not the same for everyone, but you would … If you think about it, you’d say: ‘you’re a patient, if you can’t really do without medication.’ And I think … We are Dutch, we are much too down to earth to put labels on it, such as ‘patient’ and such, but if you listen carefully and having also experienced things myself that I think like … That I am really short of breath, that I think like, I can hardly keep going … Those are really annoying complaints. But I think we are also like, at the end of the day it’s having a pause and then you keep on going and tomorrow will be a better day. Hoping for some rain in the evening, indeed [name participant]. (focus group 1, online).

In addition to aforementioned aspects, participants also discussed social issues related to their allergies. During the COVID-19 pandemic, having hay fever became particularly burdensome. Constant sneezing made them feel uncomfortable, and they frequently had to undergo COVID-19 testing to make sure the symptoms were not due to the virus, adding an extra layer of inconvenience and concern. However, despite experiencing symptoms, participants generally expressed a reluctance to cancel social events, demonstrating their determination to participate despite their allergies. Furthermore, they reported that their loved ones showed significant support and understanding regarding their allergy situation, providing them with a supportive network.

### Theme 2: Individual search for what does or doesn’t work

Regarding theme 2, findings from the focus groups revealed that individuals engage in their own cost-benefit consideration when deciding the type and extent of preventive efforts they invest in managing their allergies. This is influenced by several subthemes, including the uncertainty of effectiveness and individual differences in what works for each person, leading participants to rely on a history of trial-and-error experiences.

A notable aspect contributing to this uncertainty is the doubt many participants expressed regarding the efficacy of both reactive and preventive measures, including prescription drugs.

So I do use a tablet and I do notice that it is actually not quite enough, because despite that I often have itchy or at least burning eyes. Also very often it hits my voice again and keeps itching. But now I’ve been to the doctor so many times and I get eye drops and a nasal spray again and I’ve already had so much, but not really what works. (focus group 4, on location).

I’ve never really experienced much of a difference between one or the other. My feeling is always that it all just doesn’t really work, it’s just going through the motions, and maybe it’s just psychological. (focus group 1, online)

Participants exchanged and discussed various tips and tricks related to managing their allergies. For example, they shared personal insights into measures that worked for them, which helped navigate the uncertainty about the effectiveness of measures they already took for other allergies, particularly in relation to house dust mite allergies.

Regarding the distinction between preventive and reactive measures, most participants did not make a clear differentiation, but rather developed their own individual approaches to symptom management. Reactive measures were commonly employed, with participants waiting for symptoms to reach a certain level before taking further action.

It is true that the moment I get more complaints, I take some measures. So then … I’ve had a few times, earlier in the spring, that I took some more medication temporarily for a week, or if I already feel something in the morning, than I turn on the air purification in the house for an extra while. And that all helps. (focus group 2, online).

Avoiding places with high pollen concentrations was not a favoured option, as participants did not want to feel restricted by their allergies. As described earlier, they do not see themselves as patients and do not want to be confronted with their allergies all the time. However, when they were aware of high pollen concentrations, participants would opt to stay at home if possible, such as by working remotely.

There was a diversity in the use of medication among participants, both regarding prescribed and over-the-counter medication. Some relied heavily on medication, even taking it daily throughout the year, especially when managing other allergies like house dust mite allergy as well. They expressed a dependency on medication and found it essential for symptom control. On the other hand, there were individuals who preferred to minimize their medication intake, driven by a belief that taking medication should be limited to when absolutely necessary. These participants waited until early symptoms emerged before considering medication. It should be noted that concerns about side effects, particularly with prescription drugs, played a role in the decision-making process for medication use. Furthermore, the timing of medication intake is an important consideration, particularly for certain drugs that require time to exert their effect on the immune system.

I’ll go [outside], I’ll just do my thing. If it’s really bad, I sometimes take an allergy tablet, for example. Because that helps, but I really only do that when I’m really [going] somewhere, because I find it annoying to take it every day. And furthermore in the house, actually the same with that house dust mite. Just keep everything dust-free, keep it clean, wash your bedclothes often, things like that. (focus group 3, on location).

I actually started with that too, even before the hay fever season started for me, I already started taking that desloratadine, just to build it up like that. I do have eye drops now too, but that’s more for when my eyes are burning. But other than that it works pretty well for me now, if I really just start on time. (focus group 4, on location)

I did have medication and I think also the blue one, which I think [name participant] mentioned. Only that made me so incredibly tired that it actually affects me a lot. So you can’t really function. So I’ll just cope with it, I’m … I’ve been off my medication for now, I think by now …, I think for two or three years. (focus group 2, online).

### Theme 3: Information needs and uses

In our study, participants initially expressed limited perceived benefits from acquiring more information about their allergies, as they believed it would not significantly assist them in managing their symptoms. The fact that (over-the-counter) medication, as well as other measures against allergic reactions, are not specific to particular pollen contributed to this perception of limited efficacy. Participants furthermore experienced that both prescription and over-the-counter medications offered only limited relief for their symptoms.

And I have to say, in recent years I only often have Prevalin from Kruidvat^1^. That also has to do with the fact that- I wanted the medicines from the GP, which I then bought. It really has been a search, just like what [name participant] said, you get something, sometimes it works, then you’re glad it works, [then] the GP or the pharmacy switches to another brand, you get something else, it doesn’t work again. Well, fiddled around with it for years but never found anything good. (focus group 1, online)

As described in theme 1, there is a tendency for participants to trivialize their allergy-related concerns, emphasizing a desire not to let allergies dominate their lives or consume excessive time and attention. This affects their motivation to receive information about pollen, as providing more information also draws more attention towards their allergies. This tendency was particularly pronounced when participants experienced limited effectiveness of measures aimed at controlling their symptoms.

You know, and that may also partly have to do with the thought that you then think, why would I want to know more? It doesn’t get any better. And then you don’t want to be confronted with that and you think like, it’s OK as it is. (focus group 2, on location).

This mindset, combined with mixed experience with healthcare providers, particularly GPs, resulted in a perception that the healthcare system offered insufficient assistance and information. However, participants reported more positive experiences with specialists, especially with ear, nose and throat (ENT) specialists or allergists.

Because it actually happened to me when I happened to get another GP and he says, why weren’t you sent to the ENT earlier by your GP here? And then everything came to light. And then it was said, why …? Says the ENT doctor, why have you never come here before? Because I can see it’s been going on with you for a long time. But then you’re not referred [to the ENT specialist], and then it is every time this pill, that pill. And then at some point then it’s just like, I always say, like breathing. It’s just part of you then, then you just accept it. But specialism is important here at the ENT doctor. (focus group 4, on location)

When, despite above reported issues, participants were looking for information related to pollen or their symptoms, they mostly used Google and websites including health websites (www.thuisarts.nl or www.ggd.nl). Participants that used Google and websites indicated being able to distinguish reliable sources from other sources of information, such as advertisements from commercial companies offering medicines. However, there were also participants who consulted Google and read the summary provided at the top of the first page without checking the source that Google uses for its summary.

“I always google everything, if I have something somewhere or something, that I think, I need to know what it could be or something. But … ” Response from another participant: “I’m not going to google health things, I’ll just look at Thuisarts.nl^2^ or something like that. I’m not going any further. You encounter all kinds of scary things.” (focus group 2, online)

Generally speaking, there was a mild interest in using pollen counts or a real-time pollen map. Their usage was primarily driven by a desire for confirmation, rather than taking preventive measures or avoiding pollen exposure. People checked pollen forecasts to validate their assumption that high pollen concentrations coincided with the symptoms they experienced, rather than using them proactively to prevent symptom onset. Consequently, as noted under Theme 2, symptom occurrence often served as a trigger for engaging in preventive behaviour and seeking information.

I might use it as a kind of confirmation or something, if I had more complaints [then usual], to see if it could indeed be explained logically. But as prevention I wouldn’t be inclined to use it so quickly, because my complaints are not so [severe]. (focus group 2, online)

Regarding future developments in tailored pollen forecasts, our findings indicated mild interest among a subset of participants. However, their willingness to use such information was dependent on certain conditions. Participants expressed a need to identify the specific pollen types to which they were allergic and desired more effective measures, such as improved medication or ‘pollen-specific’ treatments. Furthermore, they emphasized the necessity for forecasts to be reliable – in terms of geographical location, timing and pollen type – and tailored towards the individual. Finally, some preferred apps over websites, while for others this was the other way around.

Yeah, I’d find that interesting. (…) So then I’d go look it up and then I could therefore also take my medication use into account and so on and then I don’t have to … Because when my supply is finished, it’s done, and then when it gets worse again, I first have to get new ones and wait and then it’s often too late. So if I know that in advance, indeed, it would be useful if I could pick up my medication in advance and start it in advance, so that the [blood] levels have already built up somewhat. Really keep an eye on it. (focus group 4 on location)

Yes, I would like to, but now the medicines that I take now are those - I mean, I always take hay fever tablets and in fact I almost always use nasal spray. And those eye drops, that depends a bit, but that’s also kind of how much trouble I’m in. So I’m already doing a lot and then, for example, if those eye drops could prevent it or relieve complaints tomorrow, then I would … Then maybe I’d look at that […]. (focus group 1, online)

It sounds interesting if you can actually make it so person-specific. And if that actually worked in practice, I think I could get excited about it. At first I wouldn’t put an app on my phone like that on my own, but if I heard from others that it really works, then … Who knows. (focus group 1, online)

You already have so many [apps] on your phone, so it would be nice, for example, if you could sign up for a list or a push notification or something that if it comes in your area with location tracking or … (…) A type of subscription, you can indicate what you are allergic for and then you will simply receive push messages about it. That it just shows up on your phone. I’m a little tired of another app on my phone. And you already have so much and some you don’t use for a while and then they come off, then they kind of go into hibernation. And then they don’t show up anymore. (focus group 2, online)

(…), but I think it would be very useful if I had such an app and that you could see that, but also, for example, what I was just talking about, the processionary caterpillar and such. Simply, you are somewhere in an area, for example the Kralingse Bos, (…) That perhaps it works with a location that you can see, there are now many processionary caterpillars or certain grasses are now active there or - That seems to me - When I think about it that way, I think that - Maybe I would like it a little better if it became a little broader than hay fever. (focus group 2, online)

Participants indicated that the option to upload symptom scores into a real-time pollen map was an opportunity to contribute to scientific research or the greater good. Nonetheless, trivializing their symptoms or concerns about the subjectivity of their scores discouraged some participants from engaging in this type of behaviour, fearing that their scores would not be valuable to others or to scientific research.

Overall, participants expressed low information needs due to perceived limited relevance for self-management of symptoms. Seeking or receiving information about pollen allergies was often seen as confrontational, confirming their allergy status. This confrontation was commonly avoided, as individuals with severe symptoms relied on medication regardless of additional information, while those with mild symptoms preferred to wait until symptoms appeared rather than continuously using medication.

## Discussion

The present focus group study aimed to identify information needs and preferences for personalized and local pollen concentration information among adults with seasonal allergic rhinitis, based on an open exploration of their allergy self-management practices and underlying beliefs and knowledge. The results could be synthesized into three interrelated themes ‘Being ill, but not a patient: it’s bad, but you learn to live with it’; ‘Individual search for what does or doesn’t work’; and ‘Information needs and uses’. Taken together, this means that adults generally have low information needs, as they have learned to manage their allergy to the extent that they are able to function as well as possible, by trying to limit the impact of both the allergic complaints as well as the impact of self-management practices on their daily lives. Because most have been diagnosed with pollen allergy in their early childhood, people have acquired ingrained habits that allow them to deal with the most severe complaints in more or less effective ways, while accepting and also trivializing the overall burden and impact of their pollen allergy on their lives. There is large variation in the effects and side effects of medication, and as participants experience that primary care providers have limited knowledge and solutions on offer, they go on an individual search for what type of medication and other measures offer them the best package. As medication and other measures are not plant or pollen specific, people believe they do not need to know which specific pollen types they are allergic to, and they feel little need for personalized information about local pollen concentrations.

These findings are in line with the few prior qualitative studies showing that self-management practices are very diverse and individualized, and are often more reactive to (early) symptoms instead of preventive (Cvetkovski et al., 2018; Muzalyova et al., 2019). Findings also confirm earlier reports that people may trivialize the impact of allergic rhinitis (Marple et al., 2007; Muzalyova et al., 2019); do not identify as patients (Cvetkovski et al., 2018); and find allergen avoidance behaviours, such as staying indoors, impractical (Marple et al., 2007) or try to avoid taking medication as much as possible (Cvetkovski et al., 2018; Muzalyova et al., 2019). The present study extends and adds to this knowledge by showing *how* persons learned to self-manage their allergic rhinitis in ways that heavily rely on cost-benefit considerations and which can thus be considered sub-optimal from a biomedical perspective, yet are understandable from a biopsychosocial perspective. In addition, our study shows how current ingrained self-management practices reduce openness to pollen concentration information. Over the course of their lives, people have become sceptical about the accuracy and value of pollen forecasts as well as about the effectiveness of medication and the extent healthcare is able to help them. Opening themselves up for new information and self-management practices would require willingness to be confronted with being a patient (i.e. someone who suffers from a chronic condition); taking their disease more seriously which is psychologically burdensome; and trying to improve their self-management, which is potentially disappointing. Therefore, communication that aims to improve information for adults with seasonal allergic rhinitis with the goal of supporting their self-management should take into account these psychological costs and pitfalls.

### Practical recommendations

The findings of this study highlight both opportunities as well as challenges for communication that aims to improve self-management practices of adults with seasonal allergic rhinitis. Opportunities stem from the apparent lack of knowledge and awareness among participants of their own allergic profile, as well as of common allergic plant species and their flowering time. Improving this knowledge may be supportive of taking timely preventive action to avoid exposure to atmospheric pollen peaks. A related opportunity is to address people’s somewhat limited knowledge on the workings and effective use of medication. Additionally, some people express an interest in tailored pollen predictions, so they can take preventive measures to avoid exposure to expected pollen peaks and thus have fewer of no symptoms. Their current way of using pollen predictions, which is as a confirmation and explanation of their complaints, could act as an entry point to ‘tempt’ people to use pollen predictions that support them to avoid the impact of expected pollen peaks. Finally, general practitioners (GPs) may adopt a proactive approach in ensuring that those presenting with seasonal allergic rhinitis are adequately tested and diagnosed. After diagnosis, the GP could disseminate comprehensive information on allergies and specific allergy types, and the effectiveness of medication and other preventive measures. By actively engaging in patient education, GPs can empower individuals to better understand their allergies and make informed decisions about preventive measures (Cvetkovski et al., 2024).

However, some challenges arise in this respect, as many people do not consider themselves as patients, and mostly avoid being confronted with their condition; trivialize their complaints; and have ingrained habits of managing their allergy that strike a balance between effectiveness and efficiency. As such, the target group is not necessarily open to information, and information may not be attractive as it can be confronting and confirms the personal and societal impact of being allergic. One possibility to deal with this is to present comprehensive information on a website that is easily found through common search engines and which is also used and recommended by healthcare providers and pharmacies. This is not a ‘quick fix’, as it takes time to gain awareness of and trust in such an information source, among both adults with seasonal allergic rhinitis and professionals.

### Future research

Future studies could validate our findings in larger, and ideally representative samples of adults with seasonal allergic rhinitis, for example, with survey questionnaires. Such an approach is able to quantify relationships between the beliefs that were identified in the present study and subsequent information-seeking and self-management practices. This might provide additional insights into the most important and changeable beliefs that could effectively be addressed using targeted communication strategies (Eldredge et al., 2016).

Another, complementary approach is developing and testing components for effective communication with adults with seasonal allergic rhinitis, and preferably also with healthcare providers. Through focus groups, but also using experimental designs, specific messages, even personalized, could be tested for attractiveness, trustworthiness and effectiveness.

### Strengths and limitations

This study is among the first to explore self-management and information practices of adults with seasonal allergic rhinitis. The broad exploration of this topic, and the open and committed way the participants discussed how they dealt with their allergy can be seen as strengths. This was observed both for online and face-to-face focus groups, showing similar discussion dynamics and content. Another strength is the diversity within our study sample (see Table 1) with regard to the severity of allergic complaints, educational level and geographical spread within the Netherlands. However, in-depth information about the exact type of allergies, comorbidities and current treatments was not available for our sample. This can be seen as a limitation in the background information of the sample, together with not having specific medical inclusion or exclusion criteria for including participants. There were two reasons for not collecting medical information. First, the availability of medical information as a precondition would hamper recruitment and selection, as this type of information is not available or accessible. Both patients and their healthcare providers are often not aware of the exact allergy types they suffer from, because they were never formally tested, or because testing occurred a long time ago. Second, we were interested in a diverse sample, in order to examine the widest possible range of self-management and information needs that may exist within this population. Any exclusion criteria based on allergy type would only result in potentially limiting that range. Finally, although focus group discussions started by requesting participants to draw everything they were allergic for, this was not intended as a comprehensive anamnesis but to give researchers and participants an idea of the diversity within the group, to activate thinking about allergies and kick-start the group discussion.

Saturation was notable for the themes ‘Being ill, but not a patient: it’s bad, but you learn to live with it’, and ‘Individual search for what does or doesn’t work’. However, a limitation of our study might be that the theme of ‘Information needs and uses’ was more difficult to explore. On the one hand, this is a useful result, as participants expressed not being very open to information, or only conditionally. On the other hand, as this theme was explored towards the end of the focus group discussions, some of the focus of participants and researchers may have waned somewhat. Finally, only two participants showed up for one out of the two focus groups that were held on location. The other participants did agree to participate by phone, but unfortunately did not show up, even while an additional attempt was made to call them and persuade them to come at the evening of the focus group. However, we decided to proceed with the two participants and together we were still able to have an interesting and lively discussion. Moreover, similar topics and themes emerged during this discussion as compared to the other ones, indicating that the limited group size seemingly did not affect the content of what was discussed.

## Conclusion

Adults with seasonal allergic rhinitis manage their complaints based on what they see as the costs and benefits of self-management, even if it is not the most medically effective approach. They may not be open to new information, such as personalized pollen forecasts, because they don’t consider themselves as ‘patients’, downplay their symptoms, and have established ways of managing their allergies. Some people have become sceptical about medications and healthcare’s ability to help them. On top of that, processing information about seasonal allergy means acknowledging their condition and working on self-management, which can be emotionally difficult. Although challenging, opportunities include improving people’s knowledge about their allergies, common allergens and how medications work. Some people do express an interest in personalized and location-specific pollen forecasts to help them avoid symptoms, especially if it matches their media use and preferences. As a start, offering comprehensive information about seasonal allergy on a website from a known and trustworthy source could be a start, but it will take time to gain trust among the population and professionals.

## Supplemental Material

sj-docx-1-hpq-10.1177_13591053241272150 – Supplemental material for Self-management and information needs of adults with seasonal allergic rhinitis in the Netherlands: A focus group study Supplemental material, sj-docx-1-hpq-10.1177_13591053241272150 for Self-management and information needs of adults with seasonal allergic rhinitis in the Netherlands: A focus group study by Bob C Mulder, Marise J Kasteleyn, Lisbeth Hall, Arnold JH van Vliet and Letty A de Weger in Journal of Health Psychology

## Notes

1. A pharmacist selling over the counter medication.

2. Website about health and illness, developed and maintained by the Dutch College of General Practitioners.
