# Supplementary material for: Self-management and information needs of adults with seasonal allergic rhinitis in the Netherlands: A focus group study
Source: J Health Psychol. 2024 Aug 22;30(6):1291–305. doi: 10.1177/13591053241272150 (PMC12053067; doi:10.1177/13591053241272150)
Supplement: sj-docx-1-hpq-10.1177_13591053241272150 – Supplemental material for Self-management and information needs of adults with seasonal allergic rhinitis in the Netherlands: A focus group study [file sj-docx-1-hpq-10.1177_13591053241272150.docx]

Appendix I: Topic list for moderating the focus group discussions

*Nature and severity of the pollen allergy*

Opening question: how have you experienced the pollen season so far?

- Current complaints
- When and how participants have been tested for allergies
- Impact of the complaints on quality of life
- Occurrence of food allergies and/or asthmatic complaints
- Knowledge about the specific pollen they are allergic to
- Knowledge about the period of complaints/flowering of the plant
- Recognizing the flowering of the plant
- Knowledge about locations of the plants that cause symptoms
- The extent to which people identify themselves as 'patient' or as having an illness

*Treatment/prevention/dealing with complaints*

Opening question: How do you deal with the allergy? What can be done to reduce and/or prevent complaints?

- Medication use (duration, timing (at the time of complaints or before)
- Avoid exposure to pollen (stay indoors, walk around/cycling)
- What does/doesn't work to alleviate symptoms
  - including perceived reasons why it works/not works.
- Tips for other people dealing with pollen allergies
- Social influences; including social support, social pressure, and being judged by others

*Participant needs to better deal with complaints/reduce complaints*

Opening question: What sources of information about (dealing with) the allergy do you use, and how do you rate their trustworthiness?

- How information is used in dealing with pollen allergies
- What other information is needed; what channels are preferred. E.g.:
- Websites
- Apps
- General practitioner/specialist
- Pharmacy
- Other

*Interest in (tailored) pollen counts and forecasts*

Opening question: To what extent do you use pollen counts/pollen forecasts?

- View on pollen forecasts
- View on regional pollen counts
- Using pollen forecasts to plan activities for the next day or to take your medication earlier.
- Motivation to enter symptom scores to get more detailed information.

Closing question: what other solutions that have not yet been discussed would participants like to see?
